# Supplementary material for: Seminoma and Embryonal Carcinoma Footprints Identified by Analysis of Integrated Genome-Wide Epigenetic and Expression Profiles of Germ Cell Cancer Cell Lines
Source: PLoS One. 2014 Jun 2;9(6):e98330. doi: 10.1371/journal.pone.0098330 (PMC4041891; doi:10.1371/journal.pone.0098330)
Supplement: File S1 — ZIP file containing DMRforPairs output for significant regions. Please start from the html files. (ZIP) [file pone.0098330.s008.zip › figures/5392.pdf]

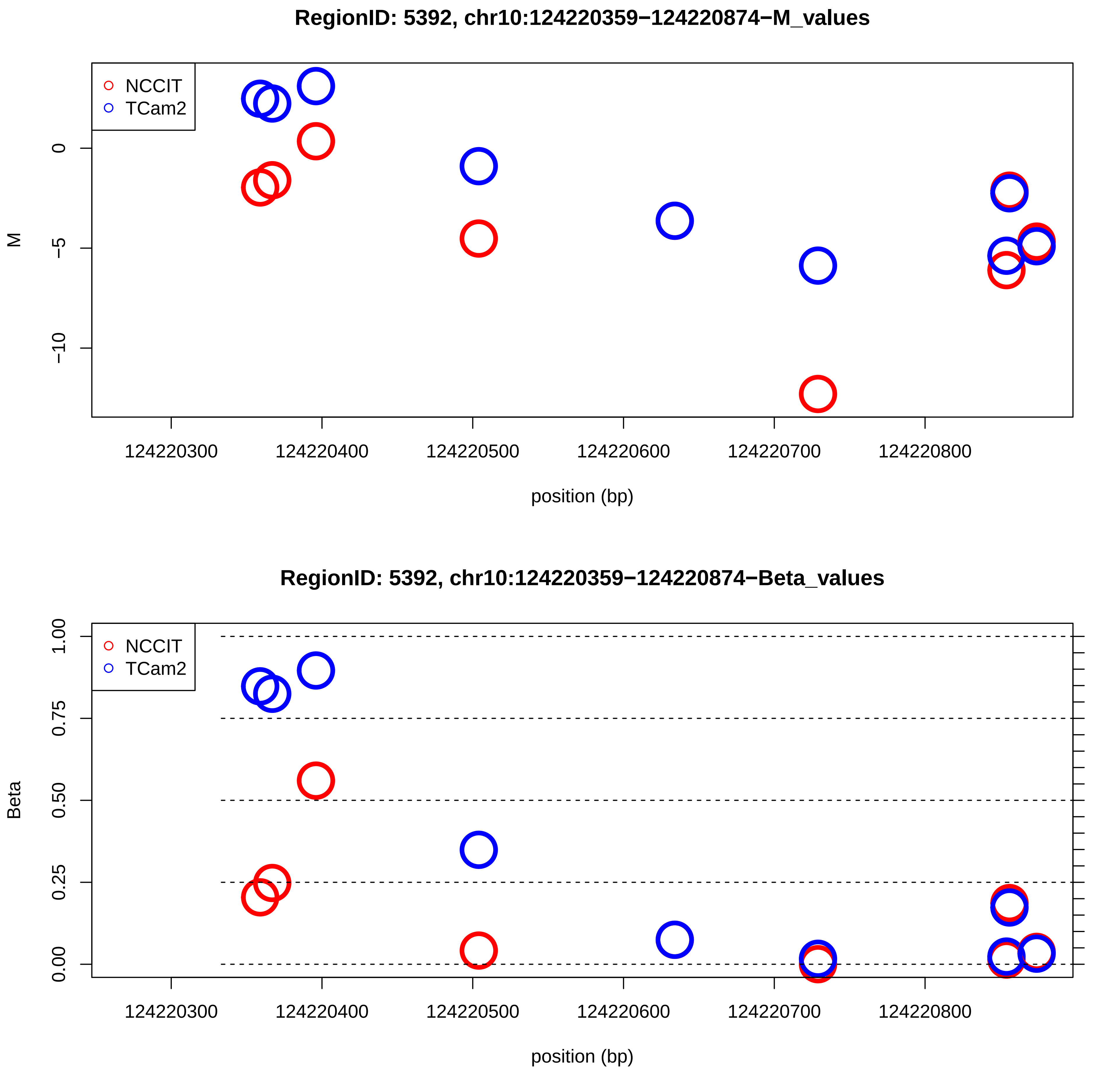

RegionID: 5392, chr10:124220359−124220874−M_values
NCCIT
TCam2
M
0
−5
−10
124220300
124220400
124220500
124220600
124220700
124220800
position (bp)
RegionID: 5392, chr10:124220359−124220874−Beta_values
NCCIT
TCam2
Beta
0.00
0.25
0.50
0.75
1.00
124220300
124220400
124220500
124220600
124220700
124220800
position (bp)
